# Novel idea generation in social networks is optimized by exposure to a “Goldilocks” level of idea-variability

**DOI:** 10.1093/pnasnexus/pgac255

**Published:** 2022-11-24

**Authors:** Raiyan Abdul Baten, Richard N Aslin, Gourab Ghoshal, Ehsan Hoque

**Affiliations:** Department of Computer Science, University of Rochester, Rochester, NY 14620, USA; Haskins Laboratories and Department of Psychology, Yale University, New Haven, CT 06520, USA; Department of Physics and Astronomy, University of Rochester, Rochester, NY 14627, USA; Department of Computer Science, University of Rochester, Rochester, NY 14620, USA

**Keywords:** self-organizing social networks, creativity, social influence, divergent thinking

## Abstract

Recent works suggest that striking a balance between maximizing idea stimulation and minimizing idea redundancy can elevate novel idea generation performances in self-organizing social networks. We explore whether dispersing the visibility of high-performing idea generators can help achieve such a trade-off. We employ popularity signals (follower counts) of participants as an external source of variation in network structures, which we control across four conditions in a randomized setting. We observe that popularity signals influence inspiration-seeking ties, partly by biasing people’s perception of their peers’ novel idea-generation performances. Networks that partially disperse the top ideators’ visibility using this external signal show reduced idea redundancy and elevated idea-generation performances. However, extreme dispersal leads to inferior performances by narrowing the range of idea stimulation. Our work holds future-of-work implications for elevating idea generation performances of people.

Significance statement Research on group creativity typically overlooks how real-life social networks (e.g. among academics or musicians) change with time. In these networks, a small fraction of high-performing ‘creative superstars’ achieves high visibility as many peers seek inspiration from their ideas. This can help the superstars inspire novel ideas in their peers but can also introduce redundancy in those inspired ideas. We show that if people spread out who they take inspiration from, it can help reduce redundancy in the inspired ideas. However, taking inspiration from peers with subpar ideas fails to inspire novel ideas in people. In our study design, the inspiration sources of people’s ideas are traceable, and the effects of spreading out who people take inspiration from are directly observable.

## Introduction

Creative ideas are generated by individuals, but creativity rarely emerges from a social vacuum. Our social embeddings exert a strong influence on the idea inspirations we find, just as it does on the information we receive, the decisions we make, and even the beliefs we hold (1–6). For example, novel idea generation hardly ever happens in isolation in academia, as researchers take inspiration from each other all the time. The social network dynamics of who takes inspiration from whom can then critically impact the idea-generation performances of a population. Importantly, human networks are known to self-organize temporally, as people make and break ties with others based on cues such as skill/competence, success, prestige, and self-similarity (7–9). The question is, in a self-organizing social network, what kind of connection structures can best support the social stimulation of novel ideas in people?

Unfortunately, despite its ubiquity, the temporal self-organization characteristic of social networks has received rather little research attention when it comes to novel idea-generation performances of humans. Recent explorations suggest two opposing factors that influence idea generation performances in this setting (10,11). On the one hand, people selectively seek inspiration from (or "follow", as referred to henceforth) the high-performing peers in their networks. This positively benefits people’s idea generation performances, as the highly novel ideas can help stimulate further novel ideas in the followers by enabling them to make remote connections between concepts that they originally didn’t think of (12,13). This process also makes the high performers increasingly visible and central in the network as more people seek inspiration from their ideas with time. In our example of academia, the distribution of publications and citations at an individual level is extremely skewed (14), allowing some ‘academic superstars’ to outshine others in terms of visibility and contribution. This small fraction of academic superstars can be an irreplaceable source of ideas to their peers (15). Similar skewed distribution of visibility and prominence emerges in other creative domains as well (16). However, there is a drawback to such performance-based self-organization of networks. As a consequence of the increasingly skewed visibility and centrality of a small fraction of high-performers, the followers’ sources of inspiration gradually become overlapping. This narrowing of stimuli idea-space can ironically inspire redundant ideas in people (10)—a rather undesired outcome.

To best support idea generation outcomes, we want people to avail the stimulation advantages of "following" the high-performing ideators as much as possible, but not to the point where redundancy starts to stifle novel idea generation in the social network. One potential solution to reducing idea redundancy is to spread out who people seek inspiration from by dispersing the skewed follower counts (in-degree centralities) of the top-performing ideators. However, in the extreme case, a full dispersal of in-degree centralities—where every ideator in the social network enjoys the same number of followers—can backfire, since such extreme dispersal can expose many people to subpar stimuli and hurt the stimulation of novel ideas in the first place. We hypothesize that a middle ground of partially dispersing the in-degree centralities—where the follower counts of the highest-performing ideators are diffused among good-performing ideators but not among poor-performing ideators—can reduce idea redundancy while maintaining high stimulation, and thus lead to superior idea generation performances. The study design to test the hypothesis is explained below.

### Experiment design and rationale

There are several challenges inherent in this line of inquiry, e.g., unambiguously tracking the links between people’s ideas and the respective inspiration sources, identifying what cues guide the network connections (i.e., inspiration links) to evolve with time, and detecting whether network self-organization patterns have any impact on the idea generation performances of people.

To address these, we use a recently developed experimental approach that facilitates conducting randomized controlled experiments in the temporal social network setting (10,11). The experiment design builds on ego-centric networks, which specifically map the social inspiration ties from the perspective of a single person (an “ego”) to their peers (“alters”). Here, the ego is the focal person whose idea generation performance we care about, and the alters’ ideas provide the stimulation sources for the ego to take inspiration from. To enable a randomized controlled setup, independent samples of egos are taken for each study "condition," but the set of alters (and their respective ideas) is held constant across all study conditions. Then, modifying only one modality of social signal/cue of the alters across study conditions enables us to test that modality’s influence on the network connection choices and associated idea generation performances of the egos in the respective conditions (11).

**Fig. 1.**
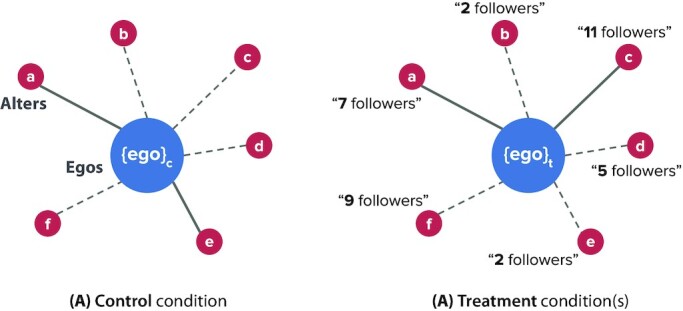
Intuition behind the study design. Our study has four conditions, but we show two conditions here for illustration. The set of alters (and their respective ideas) is kept constant across all conditions, and each condition has an independent sample of egos. The {ego} notation denotes that all egos in a condition receive the same information about the fixed alters. (A) In the control condition, the egos see only the ideas of the alters when making their connection choices. The solid lines illustrate that each ego forms ties with two alters out of the six. In our study, condition C1 constitutes a control condition. (B) In the treatment condition(s), the egos are shown the follower counts (real or fake) of the alters in addition to the alters’ ideas. We expect the egos’ connection choices to be biased by this additional popularity signal, which can allow us to manipulate the in-degree centralities of the alters to test our hypothesis. We have multiple treatment conditions (C2 to C4), where the shown follower counts are manipulated differently across conditions.

In our study, we leverage popularity signals (i.e., follower counts) of the alters as the external source of variation in the ego-centric network structures. Popularity signals have previously been reported to socially influence cultural, economic, and academic networks (16–18). People can conform to social influence when they assume others to be better informed than they are or believe that the majority’s pick is correct (2,16,19,20). Here, the alters’ popularity signals (follower counts) are used as the vehicles of social influence on the egos. Such signals can trigger anchoring biases, where a higher follower count shown on the screen can anchor an ego with an inflated impression of the alter’s idea generation performance, and vice versa (21–23). This, in turn, can bias the egos’ choices of social sources of inspiration. We use popularity signals to bias the egos’ connection choices differently across various study conditions, which allows us to systematically assess how changes in the alters’ in-degree centralities correspond to the individual and collective idea generation performances of the egos. Fig. 1 explains the intuition behind the study design.

We use a custom web interface to conduct our randomized experiment in the virtual laboratory. First, we adopt a text-based divergent thinking task where the participants generate alternative use ideas for five common objects in five successive "rounds". We randomly assign the participants either of two roles: (i) alters, whose ideas are recorded first to be used as stimuli, and (ii) egos, who take inspiration from the alters. In each round, the egos first generate ideas independently (turn-1); then see the ideas of the alters they are following and submit any inspired ideas (turn-2); and finally, rate all of the alters’ ideas and follow/unfollow them. This setting allows us to explicitly track the inspiration links as the networks evolve (more details in the "Materials and Methods" section). Second, the ideas of the same alters are shown to the egos of four different conditions, where we only modify the alters’ follower counts as shown to the egos. This creates multiple, parallel histories of network evolution across conditions. Third, we repeat the entire process for four independent "trials," each with its own set of participants. This ensures that the results do not overfit to the specific alters and helps us leverage a rich variation in the data for deriving the insights.

**Fig. 2.**
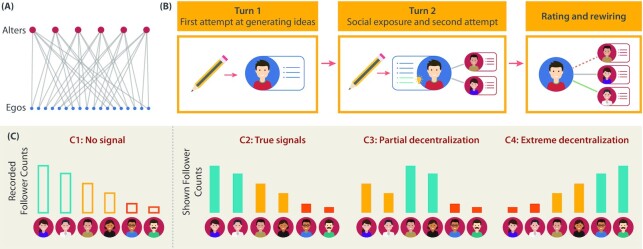
(A) The initial network structure with alter and ego nodes. Each ego is connected to two alters out of six. (B) Protocol for each of the five rounds of idea generation (with one idea-generation prompt in each round). In turn-1, an ego (blue circle) generates ideas independently. In turn-2, they view the ideas of the two alters (red circles) they are following and submit any new ideas inspired by the stimuli. Then, they rate the ideas of all six alters and update which two alters to follow in the next round. (C) The four-study conditions. The top, middle, and bottom tiers of alters, as recorded from C1, are respectively shown in teal, mustard, and orange bars.

In the first condition ("C1: No Signal"), no follower count of the alters is shown to the egos at all. From here, we record the alters’ true/unbiased counts of followers in each round and rank the alters into three tiers of popularity (top, middle, and bottom) based on their total follower counts in the five rounds. In the "C2: True Signals" condition, we show the egos the true follower counts of the alters in each round, as recorded from C1. In the "C3: Partial Decentralization" condition, we swap the follower counts of the first two tiers of alters, leaving the follower counts of the bottom tier alters untouched. The ideas of the alters are kept unchanged. This implicitly makes the second tier of alters appear more popular (and the first tier less popular) than they actually are in C1. Based on the social influence literature, we expect that the in-degree centralities of the top-tier alters will be diffused among the middle-tier alters in this condition, but not among the bottom-tier alters. Our hypothesis is that the C3 condition will help reduce redundancies in the egos’ ideas while preserving sufficient stimulation benefits. In the "C4: Extreme Decentralization" condition, the follower counts are shown in the reverse order of the alters’ true popularity ranking. This condition makes the alters least popular in C1 appear the most popular, and vice versa, to bring even the least followed alters into the limelight (Figs. 2A to C). As explained earlier, we expect that such extreme dispersal of centrality may hurt the stimulation of novel ideas in the egos.

## Materials and methods

This study was approved by the IRB of the University of Rochester, USA. All participants provided informed consent.

### Participants

We recruited 312 participants from Amazon Mechanical Turk who were located in the US. Each of the 4 trials had 6 alters and 72 egos. The 72 egos were split equally into the four conditions (18 egos in each). They were assigned their roles (alter/ego) and study conditions randomly. 121, 189, and 2 participants, respectively, self-identified to be of female, male, and other gender. The race distribution was: White: 238, Black or African American: 33, Asian: 21, Two or More Races: 5, other: 15. A total of 15 participants belonged to Hispanic or Latino ethnicity. The age distribution was: 18 to 24: 24, 25 to 34: 136, 35 to 44: 82, 45 to 54: 45, 55+: 25. Two-sample Chi-squared tests do not show any detectable difference in the race, gender, or age distributions across the four study conditions (*P* > 0.05 for each of the demographic variables). Thus, any observed differences among the study conditions cannot be systematically attributed to differences in the distributions of the demographic categories.

### Measures of idea generation performance

Unlike convergent thinking, which requires individuals to zero in on known correct answers (as tested in traditional school exams), divergent thinking leads people to generate numerous and varied responses to a given prompt or situation (24,25). In this paper, we focus on divergent thinking performances in terms of novel idea generation. Our divergent thinking task is based on the canonical Alternate Uses Test^a^ (26). In each of the five rounds, an everyday object (e.g., a brick) was given to the participants as an ideation prompt along with its common use (e.g., used for building). We chose the first five objects from Form B of Guilford’s test as the prompt objects in the five rounds. The participants needed to generate alternative use ideas for the object that were novel and appropriate, different from each other, and different from the specified common use. The participants were guided with examples throughout the study, as specified in the test manual.

We used two complementary metrics for quantifying the idea generation performances (of both the egos and the alters), based on previous literature. The nonredundant idea count metric quantifies how rare one’s ideas are compared to the peers’ ideas (27,28), but does not assess the ideas’ intrinsic qualities (i.e., even a great idea is not rare/novel if many people submit it). In contrast, the Creativity Quotient metric captures how semantically diverse a person’s idea-set is (29,30), but does not attempt to compare the idea-sets socially (i.e., two people having highly diverse yet identical idea-sets will achieve identically high scores). Further details are given below.

#### Nonredundant idea counts

We first discarded inappropriate submissions that did not meet the specified requirements. Since the same idea can be phrased differently by different people, we collected or "binned" the same yet differently phrased ideas together under common bin IDs. For binning the ideas, we followed the coding rules described by Bouchard and Hare (31) and the scoring key of Guilford’s test. Based on the bin IDs, an idea was marked to be "nonredundant" if it was given by at most a threshold number of people. For the alters, the threshold was set to one (i.e., if two or more alters in a given trial submit the same idea, it is considered redundant). For the egos, the threshold was set to two (i.e., if three or more egos in a condition submit the same idea, it is redundant).

The first author binned all of the ideas in the dataset, while two other research assistants independently binned the ideas of a random $25\%$ of the participants. They were shown the ideas in random order. Based on their independently curated bin IDs, we computed the total nonredundant idea counts of each participant in all five rounds together and calculated the agreements among the coders. The agreements were high both between the first and second coder (intra-class correlation coefficient, *ICC*(3, 2) = 0.93, *P* = 10^−14^, $95\%$ C.I. = [0.88,0.96], Pearson’s *r* = 0.89, *P* = 10^−15^, $95\%$ C.I.=[0.81,0.94]), and between the first and third coder (intra-class correlation coefficient *ICC*(3, 2) = 0.87, *P* = 10^−12^, $95\%$ C.I. = [0.80,0.92], Pearson’s *r* = 0.83, *P* = 10^−14^, $95\%$ C.I.=[0.72,0.90]). We used the bin annotations from the first coder in the analyses.

For collective-level analysis, we took the total number of distinct bin IDs generated by the egos in a given condition in a given trial as the collective idea generation performance marker.

#### Creativity quotient

This metric captures the semantic diversity of a person’s idea-set (29,30), but does not compare ideas socially like the first metric. If the ideas of a participant are very similar to each other, they are likely subtle variations of a small number of semantic categories. Conversely, if they are very dissimilar to each other, they likely touched many semantic categories—marking better creativity (32). To capture this intuition, creativity quotient uses an information-theoretic measure of semantic similarity derived from WordNet (33). Since the taxonomic structure of WordNet is organized in a meaningful way, we can compute the information content of each concept mentioned in a participant’s idea set from its position in WordNet. This allows us to calculate pairwise semantic similarities between concepts, which in turn allows us to calculate the overall semantic diversity of all concepts in one’s idea set. The computation procedure is summarized below (further details can be found in (30)).

Concepts are organized as syn-sets or synonym sets in WordNet (33), where the nouns are linked with ‘is a’ relationships. We removed stop-words and punctuation from the ideas before running a spell-checker on them. Next, we split the ideas into their constituting set of concepts, and converted the terms into nouns for availing the ‘is a’ relationships in the WordNet taxonomy. We then computed the information content of those concepts. The taxonomic organization of WordNet implies that concepts with many hyponyms convey less information than concepts with less number of hyponyms (34). Therefore, infrequent concepts at the leaf nodes hold more information than their abstracting nodes. The Information Content, *I*, of a concept *c* can thus be calculated as

(1) $$\begin{equation*}
\mathit{ I}(\mathit{ c}) = \frac{log\left (\frac{h(\mathit{ c})+1}{w}\right )}{log\left (\frac{1}{w}\right )} = 1- \frac{log(\mathit{ h}(\mathit{ c})+1)}{log(w)},
\end{equation*}
$$

where *h*(*c*) is the number of hyponyms of *c* and *w* is the total number of concepts in WordNet. The denominator ensures *I* ∈ [0, 1] by normalizing the metric against the most informative concept.

Given a participant’s set of ideas in turn-2 of a given round, we proceeded to compute the semantic diversity as follows. Between every pair of concepts in the idea-set, *c*_1_ and *c*_2_, we calculated the semantic similarity (35) as

(2) $$\begin{equation*}
\mathit{ sim}(\mathit{ c_1},\mathit{ c_2})= 1-\left (\frac{I(\mathit{ c_1})+I(\mathit{ c_2})-2\times \mathit{ sim}_{MSCA}(\mathit{ c_1},\mathit{ c_2})}{2} \right ),
\end{equation*}
$$

where *sim*(*c*_1_, *c*_2_) is a function of the information overlap between the two concepts, *sim_MSCA_*(*c*_1_, *c*_2_). This overlap, in turn, is computed using the information content of the Most Specific Common Abstraction (MSCA) that subsumes both of the concepts

(3) $$\begin{equation*}
\mathit{ sim}_{MSCA}(c_1,c_2) = \max _{c^{\prime }\in S(c_1,c_2)} \mathit{ I}(c^{\prime }),
\end{equation*}
$$

where *S*(*c*_1_, *c*_2_) is the set of concepts subsuming *c*_1_ and *c*_2_.

Given the pair-wise concept similarities, we computed the multi-information, *I_m_*, as the shared information across the idea set. We crafted the max spanning tree from the network of concepts and their pairwise similarity values. We summed over the edge weights in the max spanning tree to get *I_m_*. Finally, we calculated *Q* as

(4) $$\begin{equation*}
\mathit{ Q} = \mathit{ N}-\mathit{ I}_m,
\end{equation*}
$$

where *N* is the total number of concepts in the person’s idea set.

To compute collective-level creativity quotients, we collected in a bag-of-words document all of the ideas generated in turn-2 of each round by all of the trial-wise egos and calculated the creativity quotient of the document using the same procedure as above.

### Procedure

We randomly placed the egos in the network structure shown in Fig. 2A, which was used as the initial network structure to assign each ego to follow two alters out of the six in the trial. Each of the five rounds of idea generation had three steps. First, the egos generated ideas independently on the prompt object (turn-1). Second, the egos were shown the ideas of the two alters they were following. They could submit any new ideas that were inspired by the alters’ ideas (turn-2). Third, the egos were shown the ideas of all six alters, which they rated on novelty on a five-point Likert scale (1: not novel, 5: highly novel). Then the egos could optionally choose for themselves which two alters (out of six alters) to follow in the next round (Fig. 2B). Since every ego rated the ideas of all six alters before making the following/unfollowing choice, they all had the same global knowledge about the alters’ ideas. Thus, we do not anticipate any bias in the egos’ following patterns coming from varied exposure to the alters. Only the alters’ follower counts were shown differently across conditions. Each ‘tier’ (ranked from C1) had two alters (Fig. 2C). The study interfaces are shown in SI Figs. S1 to S3.

The egos were given 3 minutes in each of turn-1 and turn-2 to generate ideas on the given object. They were forbidden to re-submit their followee alters’ ideas exactly, and were told that their nonredundant idea counts would contribute to their performances. The egos were also informed of a short test to be held at the end of the study, where they would need to recall ideas shown to them. This was in place to ensure the participants’ attention to the stimuli ideas, which is known to have positive stimulation benefits (36–39). As the egos (optionally) followed/unfollowed alters in each round, they were required to submit the rationale behind updating/not updating their links. This helped ensure the accountability of the egos in making their choices, which is known to raise epistemic motivation and improve systematic information processing (40,41). The participants were paid $10 upon the completion of the tasks, as well as a bonus of $5 if they were among the top five performers in their trials.

### Capturing dispersion of the alters’ in-degree centrality

The popularity of an alter *i* is defined by his/her share of followers, $m_i = d_i/ \sum _{k=1}^{S}d_k$, where *d_i_* is alter *i*’s follower count in a given round and *S* is the number of alters in the trial. The Gini coefficient is given by

(5) $$\begin{equation*}
G = \frac{\sum _{i=1}^S \sum _{j=1}^S |m_i - m_j| }{2S \sum _{k=1}^S m_k}.
\end{equation*}
$$

This represents the average difference in follower counts between pairs of alters, normalized to fall between 0 (complete equality or decentralization) and 1 (maximum inequality or centralization).

### Capturing overlaps between idea-sets

The Jaccard Index captures the overlap between two idea sets, *A* and *B*, as

(6) $$\begin{equation*}
J(\mathit{ A},\mathit{ B})=\frac{|\mathit{ A}\cap \mathit{ B}|}{|\mathit{ A}\cup \mathit{ B}|}.
\end{equation*}
$$

If *A* = *B* = ∅, we take *J*(*A, B*) = 1. The idea sets consist of bin IDs from the nonredundant idea count calculations.

## Findings

### People preferentially take inspiration from the high-performing peers in their networks

We test how the alters’ idea generation performances affect the egos’ inspiration link choices. We limit this analysis to the C1 condition, where no popularity signal of the alters is shown to the egos—allowing us to track unbiased or "true" following patterns of the egos. To this end, we first compute the nonredundant idea counts and the creativity quotients for each alter in each round. Then, using linear regression, we explore how the two performance metrics correspond to the alters’ popularity/follower counts. As the dependent variable *y_i_*, we take the fraction of egos connected to an alter *i* at the end of the fifth round. By the end of the fifth round, the egos are fully informed of the alters’ past performances, so we can expect their final choices to reflect that knowledge. The independent variables are: (1) the relative number of nonredundant ideas, $u^{\prime }_i = \frac{u_i}{\sum _i u_i}$, and (2) the relative creativity quotient, $Q^{\prime }_i = \frac{Q_i}{\sum _i Q_i}$ of alter *i*. Here, *u_i_* and *Q_i_*, respectively, denote the total number of nonredundant ideas and total creativity quotients of alter *i* in all five rounds together. We take the relative performance of the alters with respect to other alters in a given trial, since the egos could only choose from a fixed pool of alters. Mathematically,

(7) $$\begin{equation*}
y_i = \beta _0 + \beta _1 Q^{\prime }_i + \beta _2 u^{\prime }_i.
\end{equation*}
$$

Table 1 summarizes the results. We first test the independent variables separately using univariate regression. Using *Q*^′^ as the lone predictor gives adjusted-*R*^2^ = 0.58 (Model 1), while *u*^′^ alone leads to adjusted-*R*^2^ = 0.52 (Model 2). The positive and significant standardized regression coefficients in both models show that the two independent variables are systematic predictors of the dependent variable on their own. A higher idea-generation performance of an alter leads to a higher follower count in both models. Moreover, taking the two independent predictors together in Model 3 gives an improved adjusted-*R*^2^ = 0.69. This suggests that the two metrics hold complementary information in explaining the variation in the dependent variable, and thus do a superior job in the prediction when taken together.

**Table 1.** Regression results of predicting the alters’ relative popularity from their relative performance markers.

Predictor	Model 1: *Q*^′^ only		Model 2: *u*^′^ only		Model 3: both *Q*^′^ and *u*^′^	
	β	*t* (std. err.)	β	*t* (std. err.)	β	*t* (std. err.)
*Q* ^′^	0.163***	5.71 (0.03)	—	—	0.11***	3.65 (0.03)
*u* ^′^	—	—	0.155***	5.05 (0.03)	0.09**	2.98 (0.03)
*R* ^2^	0.597		0.537		0.717	
Adjusted-*R*^2^	0.579		0.516		0.690	

β = standardized regression coefficient. **P < 0.01 and ***P < 0.001.

In summary, we find evidence that the egos’ connection choices are strongly captured by both the statistical rarity and the semantic diversity of the alters’ ideas: the higher performing an alter, the more ego-followers they achieve. This result corroborates the findings in (10).

### Popularity signals bias people’s following patterns

**Fig. 3.**
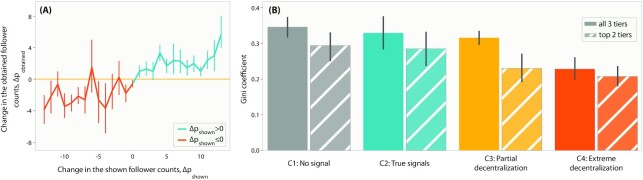
(A) Effects of changing the follower counts of the alters as shown to the egos. (B) Gini coefficient among all three tiers and among the top two tiers of the alters. Lower values denote higher decentralization. Whiskers denote 95% C.I.

Since the shown follower counts of the alters are manipulated across conditions C2 to C4, we test how the manipulations affect the following patterns of the egos in those conditions. We find that between pairs of conditions among C2 to C4, the change in an alter’s shown follower counts had (i) a significant positive correlation with the change in their obtained follower counts (Pearson’s *r* = 0.52, *P* = 10^−26^, Fig. 3A), and also (ii) a significant positive correlation with the change in the average ratings of their ideas (Pearson’s *r* = 0.11, *P* = 0.04). This suggests that the egos not only followed alters with inflated popularity signals more but also rated those alters’ ideas to be more novel. This is in agreement with previous findings that popular children can be perceived by peers as creative (18).

Since our network structure has two kinds of roles (egos/alters) of the participants, we define a "decentralized network" to be one where every alter has an equal number of followers (same in-degree centrality) (42,43). We employ the Gini coefficient metric to capture the inequality in the alters’ obtained follower counts (lower values denote higher decentralization, see the "Materials and Methods" section). Fig. 3B shows the results. C4 showed a significantly lower Gini coefficient than every other condition (post-hoc 2-tailed tests, *P* < 0.05 in all comparison cases; see SI for details), attesting that the obtained follower counts were spread out the most in this condition. In C3, the Gini coefficient among the first two tiers of alters was significantly less than the coefficient among all three tiers (2-tailed test, *P* = 0.04), showing that the decentralization happened partially among the first two tiers only, as intended. This provides validity to our choice of popularity signals as an external source of variation in the network structures. The statistical tests are elaborated in the SI text and Tables S1 and S2.

### Partial decentralization improves while extreme decentralization hurts idea generation performances

**Fig. 4.**
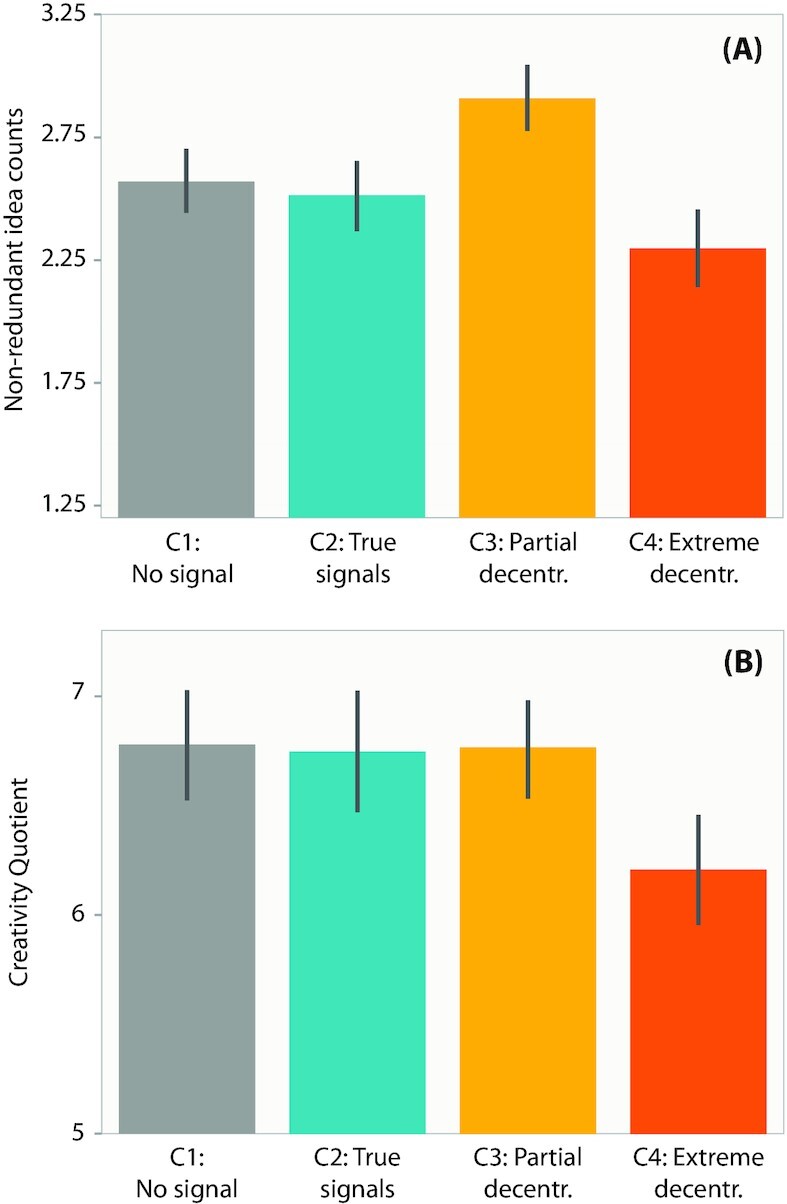
Individual-level idea generation performance comparisons across study conditions. (A) Comparison of nonredundant idea counts. (B) Comparison of Creativity Quotients. Whiskers denote 95% C.I.

The C4 egos showed significantly lower performance than all other conditions in both of the performance metrics, while the C3 egos showed significantly higher nonredundant idea counts than all other conditions (post-hoc Wilcoxon Rank Sum tests, *P* < 0.05 in each comparison case; Fig. 4; SI Tables S3 and S4).

At a collective level, the C3 egos significantly outperformed other conditions in the total number of distinct ideas generated, while the C4 egos showed a significantly lower collective-level creativity quotient than all other conditions (post-hoc Wilcoxon Rank Sum tests, *P* < 0.05 in each comparison case; SI Figs. S4 and S5; SI Tables S5 and S6). In summary, we observe that C3 improves while C4 hurts idea generation performances. The statistical tests are elaborated in the SI.

### Idea redundancy reduces in partially decentralized networks

We shed light on the (1) ego-alter and (2) ego–ego overlaps of ideas, as measured by the Jaccard Index. We find that a lower overlap between the alters’ ideas and the egos’ turn-1 ideas is associated with an increased performance output of the egos in turn-2 (creativity quotient: Pearson’s *r* = −0.32, *P* = 0.004; nonredundant idea count: *r* = −0.11, *n.s*.). The associative theory of creative cognition suggests that exposure to ideas initially unthought of by a person can help them access remote concepts in their long-term memories and recombine those remote concepts to generate novel ideas (12,13,44). Thus, a reduced overlap between the egos’ and alters’ ideas can better inspire novel idea generation in the egos, as we confirm. Intuitively, we also find that a reduced overlap/redundancy between turn-2 ideas of ego-pairs corresponds to increased performances by the egos (nonredundant idea count: Pearson’s *r* = −0.55, *P* = 10^−7^; Creativity Quotient: *r* = −0.20, *P* = 0.08). The egos’ similar choices of alters can exacerbate such idea redundancy (10).

Importantly, C3 showed the lowest ego–ego redundancy among all conditions. This can help explain why C3 had elevated idea generation performances: by reducing redundancy in the inspired ideas. Interestingly, despite having the most spread-out visibility of the alters, C4 showed the highest ego–ego redundancy (statistical tests elaborated in the SI text and Table S7). This can be explained by the fact that C4 brought the least performing alters (i.e., subpar stimuli) to the fore, which can result in the generation of common ideas by the egos—leading to both increased ego–ego overlap and reduced idea generation performance as noted in the data.

## Discussion

Our findings contribute to the scientific study of idea-generation performances in human ensembles, particularly in self-organizing social networks. In the last six decades, a wealth of research has enriched our understanding of novel idea generation at different scales of human ensembles: individual (45), dyadic (46), crowd (47), group/team (48), and static network (49) levels. Here we focused on idea generation performances in temporally self-organizing networks, a setting that is ubiquitously characteristic of real human networks but hardly explored in the scientific literature. Naturally, people’s imagination can be stimulated by nonsocial sources as well (e.g., religion, nature, and political events). But we scoped our work to focus exclusively on the social stimulation settings, e.g., as seen among academic researchers or musicians. Our experimental setting solved the problem of explicitly tracking inspiration sources, which is difficult to do in a natural setting.

We corroborated previous findings that people systematically and overwhelmingly tend to seek idea inspiration from the highest-performing peers in their networks. Interestingly, we found that popularity signals (in terms of follower counts) can bias connection patterns in social networks, partly by biasing people’s perception of the novelty of their peers’ ideas. Social influence and conformity have widely been documented in various social settings (6), and our observations show that similar effects hold in the context of idea-generation tasks in self-organizing social networks. We used follower counts as an external source of manipulation to bias the network connection structures differently across the study conditions. This allowed us to unearth one way to tackle the previously documented bottleneck of idea redundancy in social networks: by partially dispersing the in-degree centralities of the highest-performing ideators among the good-performing ideators (but not among the poor-performing ideators). In our experiment, the Partial Decentralization (C3) condition employed such dispersal and showed the best nonredundant idea counts. This can be explained by the fact that C3 had the lowest idea redundancy among egos, which helped the egos’ ideas to stand out as "rare" when compared socially against other egos. The Extreme Decentralization (C4) condition showed the lowest creativity quotient. This can be explained by the fact that this condition spread out the in-degree centralities even among subpar alters, which could leave a negative effect on the stimulation of novel ideas in the first place. Thus, we find that dispersing the visibility of the high-performers to a "Goldilocks" extent (not too hot, not too cold, just right) can help elevate idea generation performances, whereas networks whose dispersal is too narrow or too broad tend to lead to inferior outcomes.

The findings may also inform actionable insights in practical problems. For instance, research funding is increasingly being concentrated in the hands of fewer elites. This trend can have its benefits, since elite researchers may possess the infrastructure and expertise to better utilize the funding. On the other hand, diversifying the funding allocation can enable more innovation and ensure equity in the research community. It remains a matter of active research how best to disburse research funding (50–52). As an example, one of the proposed solutions to this problem suggests flattening out or decentralizing the funding, where the wisdom of the entire scientific community may be leveraged to ensure a fair distribution of funding (53). On a similar note, our results imply that a middle ground between performance-based and fully democratized allocation can be the most rewarding for fostering novel idea generation in the broader research community—since such a middle ground can reduce idea redundancy while rewarding better performers. At an individual level, our findings alert creative practitioners (e.g., academic researchers) to the pitfalls of taking inspiration from highly visible "superstar" peers. At the same time, the results suggest a solution for the practitioners that broadening the range of who they seek inspiration from can help them reduce the redundancy pitfall.

People increasingly seek idea inspiration from peers on online social platforms like Behance, Pinterest, or ResearchGate. Our findings are relevant to such online social network settings as well, where explicit and traceable interactions among peers can take place. Our results imply that content reception in such platforms may not remain purely meritocratic but be socially influenced by the presence of popularity signals of the users (e.g., follower counts). For instance, in Behance (an online portfolio platform for artists/designers), a high "follower" count may inflate people’s perceived novelty of a designer’s work, and in turn, may lead more people to follow the said designer. It may then be useful to develop intelligent peer-recommendation systems that "spread out" which users/ideators are brought to the fore or recommended in such platforms, so that idea-redundancy in the broader online communities can be reduced. Due to the COVID-19 pandemic, many distributed teams are brainstorming using remote collaboration platforms (e.g., Zoom and Slack). Based on our findings, a balance between performance-based and fully democratized interaction structures can be ensured to reduce redundant idea generation in such distributed teams. These will leave implications for the future of work, where people will increasingly need to perform creatively in a collaborative setting (54–57).

Our study is not without limitations. All of the participants were recruited from the United States. Additional experiments with participants from other countries can help ensure the generalizability of our results. The demographic categories were not split fully evenly (e.g., there were more males than females), which can potentially bias the results. Nevertheless, in our randomized study design, there was no detectable difference in the race, gender, or age distributions across the four study conditions. Thus, the observed differences across study conditions cannot be systematically attributed to differences in these demographic category distributions. A larger sample size that is balanced on each demographic variable can increase the reliability of the results in future iterations. In our design, one constraint was that we needed to manipulate centralization among only six alters. Within this constraint, we defined the partial decentralization condition to be one where the in-degree centralities of the top tier alters (two alters out of six) were spread out among the top two tiers of alters (four alters out of six), using the externally manipulated follower count signals. Having a higher number of alters would have made it easier to conduct more graded forms of decentralization, but that would also have increased the cognitive load on the egos since the egos needed to rate the ideas of all alters before making connection choices. We expect other methods/approaches for manipulation or decentralization to generate similar results, as long as a balance can be struck between minimizing idea redundancy and maintaining idea stimulation.

We found no detectable difference in the idea generation performances of the egos in the No Signal (C1) and True Signal (C2) conditions. This could partly be due to the fact that in our study design, the egos in both conditions had full knowledge of all alters’ ideas when making the connection choices. Then, in C1, the egos’ connection choices were found to be significantly guided by the alters’ idea generation performances. In C2, the egos were shown the same ideas of the alters, along with recorded follower counts from C1—which potentially encouraged the egos in C2 to adopt the same trends of connection choices as C1 (evidenced in the Gini coefficient analysis). This similarity in network connection choices can partly explain the lack of differences in idea generation performances between the two conditions. Also, the small sample size could also have played a role in the lack of detectable differences between C1 and C2.

In our study design, the egos could make their alter choices based on only two kinds of information: the ideas of the alters (held constant across conditions) and the popularity signals of the alters (shown differently across conditions). However, in real life, additional person-specific features can dictate how people’s ideas are received by peers: not all alters are equal in their ability to influence the egos. Consider two artists who produce music in different genres, engage with different socio-cultural contexts and concerns in their music, and have different levels of personal "charisma". They may inspire their peers with new ideas differently, even sell records in different volumes. Our study setting did not incorporate such person-specific nuances. This prohibited the experimental setup from capturing the high levels of heterogeneity between how particular alters influence particular egos in real life, but the setup did allow us to study the problem scope free of potential confounding factors arising from the additional person-level nuances. Thus, we anticipate our results to hold true at a large scale where individual differences wash out.

We showed in a laboratory setting how partially dispersing the visibility of high performers can be helpful for elevating the idea-generation performances of a population. One potential way to achieve such dispersion in a real-world setting could be through top-down policy interventions (e.g., regarding grant allocations), as we discussed. In our future work, we will explore bottom-up ways of achieving such optimal connection structures by guiding individual-level connection choices in the right direction. To that end, we will investigate whether intelligent peer-recommendation algorithms can nudge online interaction patterns toward optimal structures in a data-driven manner.

## Supplementary Material

pgac255_Supplemental_File

## Data Availability

The preprocessed data and associated codebase underlying this article are available at https://github.com/ROC-HCI/goldilocks-creativity-networks.

## Footnotes

a (Guilford’s Alternate Uses Test is Copyright @ 1960 by Sheridan Supply Co., all rights reserved in all media, and is published by Mind Garden, Inc, www.mindgarden.com)

## Previous Presentation

These results have not been presented previously.

## Preprints

A preprint of this article is published at arXiv:2112.03140.
