# Supplementary material for: Novel idea generation in social networks is optimized by exposure to a “Goldilocks” level of idea-variability
Source: PNAS Nexus. 2022 Nov 24;1(5):pgac255. doi: 10.1093/pnasnexus/pgac255 (PMC9802244; doi:10.1093/pnasnexus/pgac255)
Supplement: pgac255_Supplemental_File [file pgac255_supplemental_file.pdf]

Supplementary Information for

Novel Idea Generation in social networks is optimized by exposure to a ‘Goldilocks’ level of idea-variability

Raiyan Abdul Baten, Richard N. Aslin, Gourab Ghoshal, Ehsan Hoque

Corresponding author: Ehsan Hoque
E-mail: mehoque@cs.rochester.edu

This PDF file includes:

Supplementary text
Figs. S1 to S5
Tables S1 to S7

Supporting Information Text

Extended Statistical Analysis Results

Popularity signals bias people's following patterns.

Correlation analysis. Between pairs of conditions among C2 to C4, we computed the differences in the (i) shown popularity signals (Δp_{shown}), (ii) obtained follower counts ($\Delta p_{obtained}$), and (iii) obtained average ratings of the ideas ($\Delta r_{obtained}$) of each alter in each round.

We found that the change in an alter's shown follower counts, Δp_{shown}, had (i) a significant positive correlation with the change in their obtained follower counts, $\Delta p_{obtained}$ (Pearson's $r = 0.52$, $P = 10^{-26}$, 95% C.I.=[0.44, 0.59]), and also (ii) a significant positive correlation with the change in the average ratings of their ideas, $\Delta r_{obtained}$ (Pearson's $r = 0.11$, $P = 0.04$, 95% C.I.=[0.004, 0.21]). This suggests that the egos not only followed alters with inflated popularity signals more, but also perceived those alters' ideas to be more *creative*.

Gini coefficient analysis. Since our network structure is bipartite, we quantified network decentralization based on how similar the follower counts of the alters were in a given trial. For example, if all of the alters obtained an equal number of followers, then the network would be perfectly decentralized and the egos' inspiration sources would be maximally spread out. To this end, we employed the Gini Coefficient, which is a measure of success (popularity) inequality, to capture network decentralization. A lower value of the coefficient corresponds to a higher level of decentralization.

To capture partial versus extreme decentralization, we computed the Gini coefficient separately among (A) the top 2 tiers of alters (out of 3 tiers), and (B) all 3 tiers of alters. For example, if the top 2 tiers of alters are significantly more decentralized than the full set of alters, then the network is only partially decentralized. We ran the statistical tests using a 4×2 factorial design, with 4 levels in the 'Condition' factor (C1 to C4), and 2 levels in the 'Tiers' factor (top 2 tiers, and all 3 tiers). We analyzed the data using Linear Mixed Models (LMM). Since the study consisted of 5 rounds of creative ideation with 5 different objects, we assessed the fixed effects of the two factors against the random effects of the Round IDs to account for repeated measures.

We found a significant main effect of the 'Condition' factor ($F(3, 148) = 4.8$, $P = 0.003$), and a marginal main effect of the 'Tiers' factor ($F(1, 148) = 3.71$, $P = 0.056$). The interaction between the two factors was insignificant. The omnibus test results are given in Table S1. Post-hoc pairwise comparisons showed that among all 3 tiers of alters, C4 indeed showed significantly less Gini Coefficient than each of the other three conditions (2-tailed tests, C1 vs C4: $t(148) = 4.35$, $P = 0.0007$; C2 vs C4: $t(148) = 3.72$, $P = 0.007$; C3 vs C4: $t(148) = 3.22$, $P = 0.03$). Only in C3, the Gini coefficient among the first two tiers of alters was significantly less than the coefficient among all three tiers (2-tailed test, $t(148) = -3.15$, $P = 0.037$)—confirming the partial decentralization. The full test details are given in Table S2. All of the P values are corrected for multiple comparisons using Holm's Sequential Bonferroni Procedure.

Partial decentralization improves while extreme decentralization hurts creativity. We collected the turn-2 non-redundant idea counts of the egos from different study conditions. Using the non-parametric Kruskal-Wallis test, we found a significant difference across conditions in the omnibus test (χ^2(df= 3, $N = 1440$)=38.93, $P = 10^{-8}$; each ego generates ideas for 5 rounds, thus contributing 5 data-points in the same condition and leading to a total of $N = 288 * 5 = 1440$). Post-hoc pairwise comparisons using the Wilcoxon Rank Sum test showed that C3 scored significantly higher than each of the other three conditions (C1 vs C3: $W = 55911$, $P = 0.004$; C2 vs C3: $W = 54298$, $P = 0.0001$; C4 vs C3: $W = 81338$, $P = 10^{-8}$), while C4 scored significantly lower than each of the other conditions (C1 vs C4: $W = 73250$, $P = 0.006$; C2 vs C4: $W = 71020$, $P = 0.045$). SI Table S3 shows the results.

We repeated the same analysis for the egos' Creativity Quotients in turn-2 in each round. The Kruskal-Wallis test showed a significant difference across conditions in the omnibus test (χ^2(df= 3, $N = 1440$)=14.57, $P = 0.002$). Post-hoc pairwise comparisons using the Wilcoxon Rank Sum test showed that C4 scored significantly lower than each of the other conditions (C1 vs C4: $W = 73904$, $P = 0.006$; C2 vs C4: $W = 72323$, $P = 0.028$; C3 vs C4: $W = 73996$, $P = 0.006$). SI Table S4 details the results.

As for collective-level outcomes, the Kruskal-Wallis test showed a significant difference across conditions in the omnibus test (χ^2(df= 3, $N = 80$)=25.48, $P = 10^{-5}$). Post-hoc pairwise comparisons using the Wilcoxon Rank Sum test showed that C3 outperformed each of the other three conditions (C1 vs C3: $W = 57.5$, $P = 0.001$; C2 vs C3: $W = 96.5$, $P = 0.021$; C4 vs C3: $W = 360$, $P = 10^{-5}$). C4 performed marginally lower than C2 ($W = 287.5$, $P = 0.055$). The results are shown in SI Table S5.

Next, we computed collective-level Creativity Quotients. Namely, we collected in a bag-of-words document all of the ideas generated in turn-2 of each round by all of the trial-wise egos in a given condition, and computed the Creativity Quotient of the document. The Kruskal-Wallis test showed a significant difference across conditions in the omnibus test (χ^2(df= 3, $N = 80$)=19.58, $P = 0.0002$). Post-hoc pairwise comparisons using the Wilcoxon Rank Sum test showed that C4 performed lower than each of the other three conditions (C1 vs C4: $W = 340$, $P = 0.001$; C2 vs C4: $W = 315$, $P = 0.008$; C3 vs C4: $W = 337$, $P = 0.001$). SI Table S6 shows the results.

In summary, we observed that C3 improved while C4 hurt creative performances. All of the P values reported here are corrected for multiple comparisons using Holm's Sequential Bonferroni Procedure.

Idea redundancy reduces in partially decentralized networks. We employed the Jaccard Index to quantify the overlaps between pairs of idea-sets. As the ego-ego overlap decreased, the average non-redundant idea-count showed a significantly increasing trend (Pearson's $r = -0.55$, $P = 10^{-7}$, 95% C.I.=$[-0.68, -0.37]$), while the average creativity quotient showed a moderately increasing trend (Pearson's $r = -0.20$, $P = 0.08$, 95% C.I.=$[-0.40, 0.03]$). Similarly, a decrease in the ego-alter overlap corresponded to a significantly increasing trend in the creativity quotient metric (Pearson's $r = -0.32$, $P = 0.0041$, 95% C.I.=$[-0.50, -0.10]$), while the non-redundant idea count metric also showed a slightly increasing trend (Pearson's $r = -0.11$, $P = 0.32$).

The omnibus Kruskal-Wallis test showed that the ego-ego overlaps varied significantly across conditions (χ^2(df= 3,$N =$ 80)=16.88, $P = 0.0008$). Post-hoc pairwise comparisons using the Wilcoxon Rank Sum test showed that C3 had lower ego-ego overlaps than all other conditions, with the differences with C1 and C4 being statistically significant (C1 vs C3: $W = 325$, $P = 0.005$; C2 vs C3: $W = 229$, $P = 0.441$; C4 vs C3: $W = 80$, $P = 0.006$). C4 showed the highest ego-ego overlaps among all conditions, with the differences with C3 being statistically significant and the differences with C2 being marginally significant (C2 vs C4: $W = 112$, $P = 0.072$; C3 vs C4: $W = 80$, $P = 0.006$). SI Table S7 details the results. All of the P values reported here are corrected for multiple comparisons using Holm's Sequential Bonferroni Procedure.

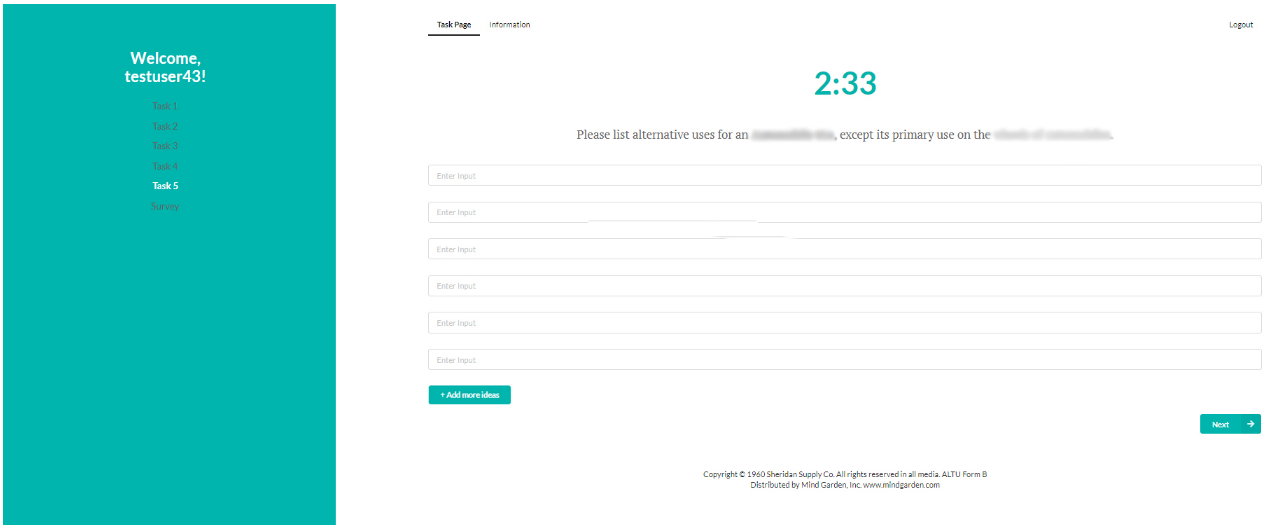

Fig. S1. Study interface: Turn-1 idea submission interface for the egos of all of the study conditions. This interface is used for recording the alters' ideas as well.

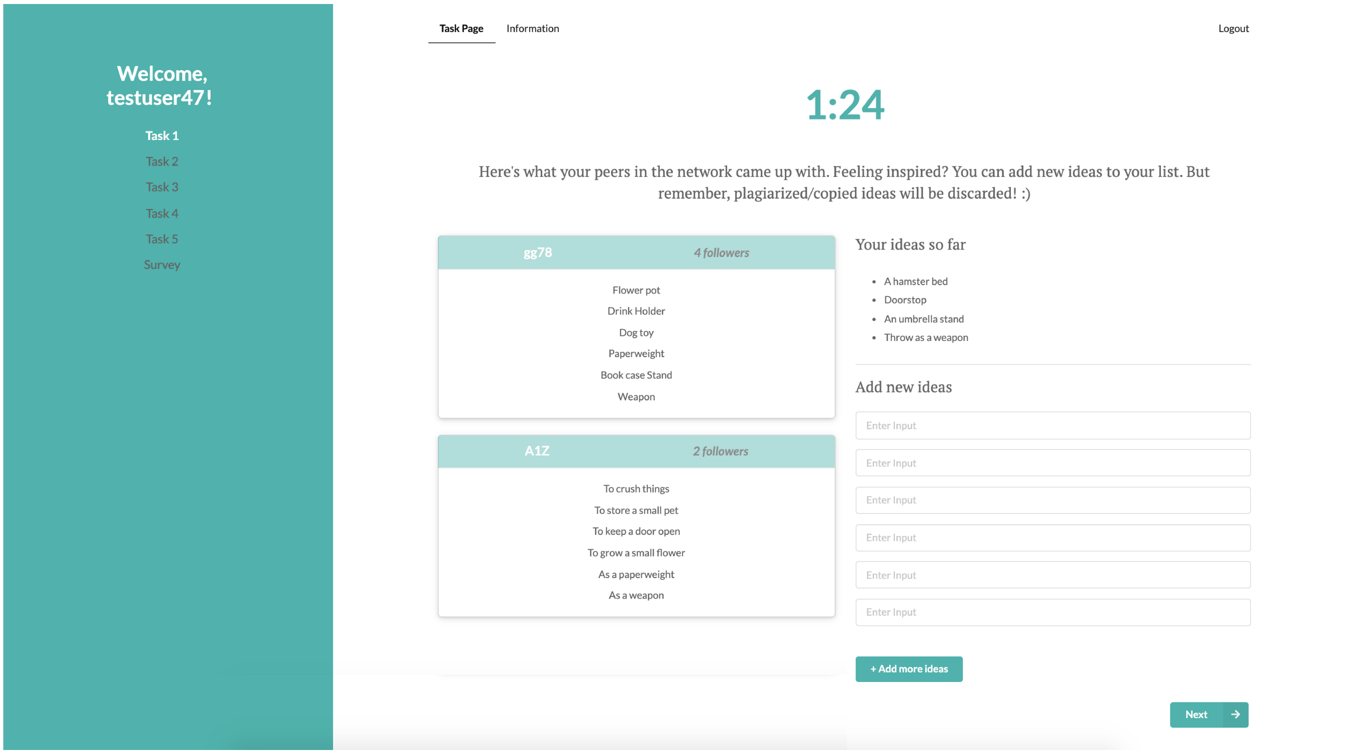

Fig. S2. Study interface: Turn-2 interface for the egos in the treatment conditions. The alters' ideas are shown on the left-side cards. In the Baseline condition, no follower count is shown.

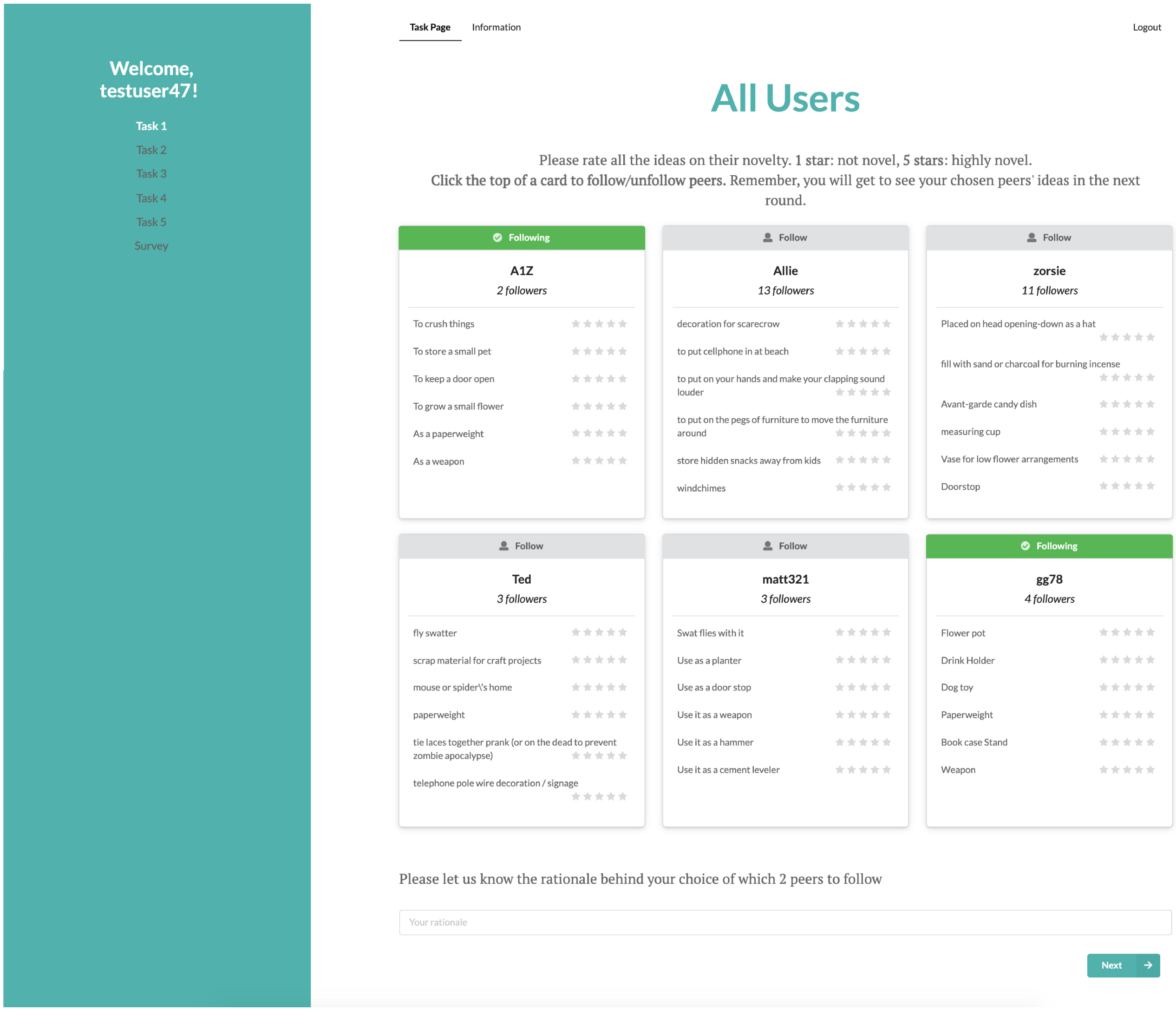

Fig. S3. Study interface: Rating and rewiring interface for the egos. In the Baseline condition, no follower count is shown.

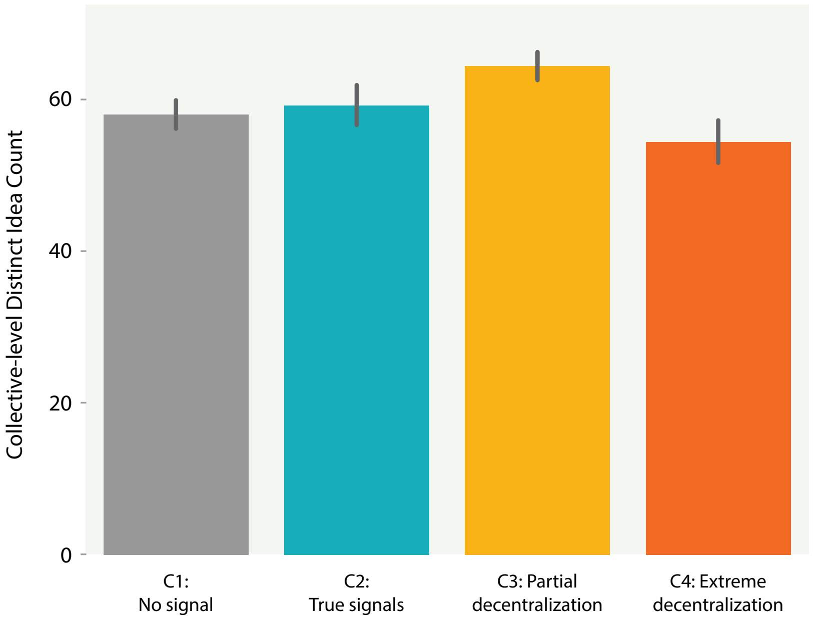

Fig. S4. Comparison of collective-level distinct idea counts in turn-2 across conditions.

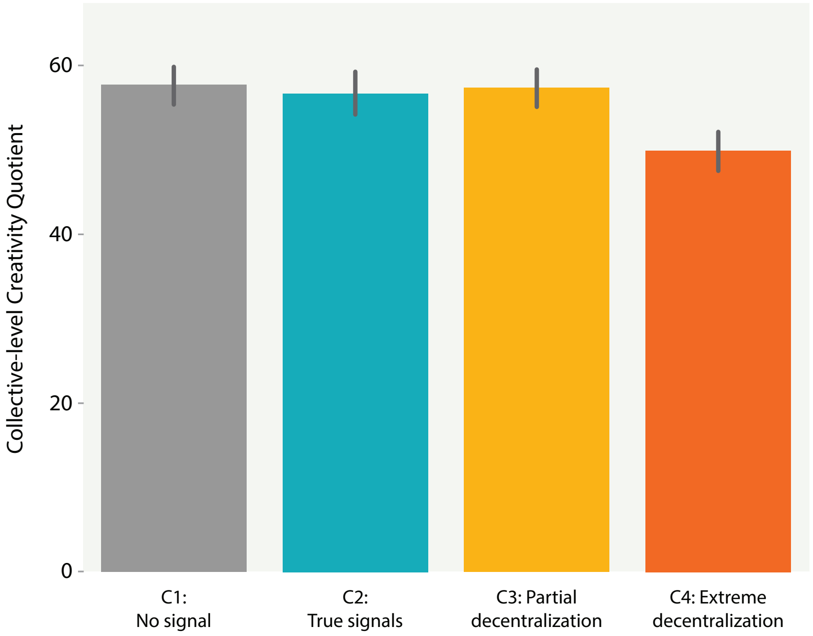

Fig. S5. Comparison of collective-level Creativity Quotients in turn-2 across conditions.

Table S1. Omnibus test results for analyzing the Gini coefficients under the factors of Study Condition (4 levels) and Tiers (2 levels). The Round IDs (denoting the 5 rounds of ideation) are taken as a random effect to account for repeated measures. The degrees of freedom are specified using the Kenward-Roger method. **$P < 0.01$, $\cdot$ $P < 0.1$.

	F	Df	Df.res	Pr($> F$)	
Study_Condition	4.8	3	148	0.003	**
Tiers	3.71	1	148	0.056	·
Study_Condition:Tiers	0.98	3	148	0.406	

Table S2. Post-hoc pairwise comparison results from the fitted model reported in Table S1. 2-tailed tests. Degrees of freedom $= 148$. P-values are adjusted for multiple comparisons using Holm's sequential Bonferroni procedure. ***$P < 0.001$, **$P < 0.01$, *$P < 0.05$.

	t-value	Std. Error	Pr($>|t|$)	
C1 (all tiers) - C4 (all tiers)	4.35	0.027	0.0007	***
C2 (all tiers) - C4 (all tiers)	3.72	0.027	0.0066	**
C3 (all tiers) - C4 (all tiers)	3.22	0.027	0.0330	*
C1 (all tiers) - C2 (all tiers)	0.63	0.027	1.0000	
C1 (all tiers) - C3 (all tiers)	1.13	0.027	1.0000	
C2 (all tiers) - C3 (all tiers)	0.49	0.027	1.0000	
C3 (top 2 tiers) - C3 (all tiers)	-3.15	0.027	0.0371	*
C1 (top 2 tiers) - C1 (all tiers)	-1.93	0.027	0.6735	
C2 (top 2 tiers) - C2 (all tiers)	-1.62	0.027	1.0000	
C4 (top 2 tiers) - C4 (all tiers)	-0.77	0.027	1.0000	

Table S3. Post-hoc pairwise comparisons among the turn-2 non-redundant idea counts of the egos across different conditions. Wilcoxon Rank Sum Test. $N = 360$ for each group. $\mu =$ group mean. P-values are adjusted for multiple comparisons using Holm's sequential Bonferroni procedure. ***$P < 0.001$, **$P < 0.01$, *$P < 0.05$.

	μ_1	μ_2	W	P-value	
C1 - C2	2.569	2.514	66803	0.461	
C1 - C3	2.569	2.908	55911	0.004	**
C1 - C4	2.569	2.297	73250	0.006	**
C2 - C3	2.514	2.908	54298	0.000	***
C2 - C4	2.514	2.297	71020	0.045	*
C3 - C4	2.908	2.297	81338	0.000	***

Table S4. Post-hoc pairwise comparisons among the turn-2 Creativity Quotients of the egos across different conditions. Wilcoxon Rank Sum Test. $N = 360$ for each group. $\mu =$ group mean. P-values are adjusted for multiple comparisons using Holm's sequential Bonferroni procedure. **$P < 0.01$, *$P < 0.05$.

	μ_1	μ_2	W	P-value	
C1 - C2	6.778	6.747	66089	1.000	
C1 - C3	6.778	6.765	65346	1.000	
C1 - C4	6.778	6.207	73904	0.006	**
C2 - C3	6.747	6.765	63770	1.000	
C2 - C4	6.747	6.207	72323	0.028	*
C3 - C4	6.765	6.207	73996	0.006	**

Table S5. Post-hoc pairwise comparisons among the total number of distinct ideas generated in turn-2 of each round by all of the egos together across different conditions. Wilcoxon Rank Sum Test. $N = 20$ for each group. $\mu =$ group mean. P-values are adjusted for multiple comparisons using Holm's sequential Bonferroni procedure. ***$P < 0.001$, **$P < 0.01$, *$P < 0.05$, $\cdot$ $P < 0.1$.

	μ_1	μ_2	W	P-value	
C1 - C2	58.00	59.50	174	0.489	
C1 - C3	58.00	64.40	57.5	0.001	***
C1 - C4	58.00	54.40	267	0.142	
C2 - C3	59.50	64.40	96.5	0.021	*
C2 - C4	59.50	54.40	287.5	0.055	·
C3 - C4	64.40	54.40	360	0.000	***

Table S6. Post-hoc pairwise comparisons among the collective-level Creativity Quotients across different conditions. Wilcoxon Rank Sum Test. $N = 20$ for each group. $\mu =$ group mean. P-values are adjusted for multiple comparisons using Holm's sequential Bonferroni procedure. ***$P < 0.001$, **$P < 0.01$, *$P < 0.05$.

	μ_1	μ_2	W	P-value	
C1 - C2	57.70	56.67	230	1.000	
C1 - C3	57.70	57.35	206	1.000	
C1 - C4	57.70	49.92	340	0.001	***
C2 - C3	56.67	57.35	181	1.000	
C2 - C4	56.67	49.92	315	0.008	**
C3 - C4	57.35	49.92	337	0.001	***

Table S7. Post-hoc pairwise comparisons among the ego-ego overlaps across different conditions. Wilcoxon Rank Sum Test. $N = 20$ for each group. $\mu =$ group mean. P-values are adjusted for multiple comparisons using Holm's sequential Bonferroni procedure. **$P < 0.01$, *$P < 0.05$, $\cdot$ $P < 0.1$.

	μ_1	μ_2	W	P-value	
C1 - C2	0.144	0.138	276	0.123	
C1 - C3	0.144	0.131	325	0.005	**
C1 - C4	0.144	0.152	152	0.398	
C2 - C3	0.138	0.131	229	0.441	
C2 - C4	0.138	0.152	112	0.072	·
C3 - C4	0.131	0.152	80	0.006	**
